# A Case Series of Permanent Dorsal Root Ganglion Stimulation

**DOI:** 10.7759/cureus.21193

**Published:** 2022-01-13

**Authors:** Taejun Lee, Emmanuel Omosor, Namath Hussain

**Affiliations:** 1 Department of Neurological Surgery, Loma Linda University Medical Center, Loma Linda, USA

**Keywords:** dorsal root ganglion stimulation, permanent drgs implantation, drgs trial, neuropathic pain, spinal cord stimulation, radiculopathy, chronic pain

## Abstract

Introduction

Neuropathic pain commonly causes high levels of pain that impairs multiple facets of the lives of patients. Multiple first-line treatments such as physical therapy and pharmacological intervention exist. Treatment refractory to these interventions may be considered for spinal cord stimulation (SCS). However, modest rates of meaningful relief leave room for improvement. Dorsal root ganglion stimulation (DRGS) has been touted to be a viable alternative solution to SCS with more specific targets and, consequently, fewer side effects. Thus, DRGS has been accepted as a better alternative to spinal cord stimulation. In contrast, we report a series of DRGS patients who had lower rates of meaningful pain relief than what was reported in the literature.

Methods

We present a series of 11 patients who underwent both DRGS trial and subsequent permanent implantation with negative outcomes (defined by ≥ 50% of pre-surgical pain) in 55% of patients. Patient records were searched for comorbidities that could potentially affect the DRGS implant (diabetes, cancer, smoking, age > 70 years old). Once delineated, the predictive value of each comorbidity for negative outcomes was estimated.

Results

Eighteen patients had a successful DRGS trial which was defined as a ≥ 50% pain reduction as well as increased ability to perform daily activities. Seven patients elected not to proceed with the permanent DRGS. Of the 11 remaining patients that had the permanent DRGS, four patients reported being completely pain-free ≥ one month following implantation, one reported a significant increase in pain improvement at four months post-operation, and six patients reported pain that was ≥ 50% of their pre-surgical pain 4-12 months following implantation.

Conclusion

In our case series, we observed a discrepancy between DRGS trial outcomes and outcomes following permanent implantation. We found that a stronger correlation may exist between worse outcomes and smoking. Older age, the presence of diabetes, and cancer had more modest associations. These comorbidities may have value as tests for predicting negative outcomes of permanent DRGS implantation. Additionally, we hypothesized that this could also be due to the presence of psychological factors which obscure the true result of the DRGS trial. Thus, we suggest that DRGS be prescribed with caution in these patient populations, and use comorbidities to test for the likelihood of negative outcomes. Limitations of this study are those that are intrinsic to a retrospective case series.

## Introduction

Neuropathic pain, especially when chronic, can significantly impact the day-to-day activities of patients as they experience high levels of pain which affect their mental health, mood, work performance, etc. In the US, the prevalence of neuropathic pain is about 7%-15% among the general population with differences in age, gender, and race. Further, ~1.5% of these patients have neuropathic pain that is refractory to the current protocol of treatment [1] outlined in the next paragraph. Thus, the management of neuropathic pain, due to its prevalence and chronicity, has a significant physical impact on the patient. 

Spinal cord stimulation (SCS), which involves inserting electrodes into the dorsal epidural space, is widely accepted as a second or third-line treatment for neuropathic pain [2]. In 2019, Bates et al. compiled existing treatment guidelines for neuropathic pain to form a comprehensive treatment protocol. In this algorithm, treatments were organized by increasing aggressiveness starting with conservative multidisciplinary care (ie. physical therapy), followed by non-opioid pharmacological intervention, interventional therapies, neurostimulation/SCS, opioid therapy, and finally targeted drug delivery [3]. SCS is recommended by the Neuropathic Pain Special Interest Group (NeuPSIG) when the chronic pain is of neuropathic origin, has a score of > 50/100 on the visual analog scale (VAS), and remains present for six months despite treatment. However, while many studies cite SCS as a form of therapy for cases that are refractory to milder treatments, the NeuPSIG gave only a weak recommendation for the use of SCS in treating failed back syndrome with radiculopathy as well as complex regional pain syndrome (CRPS) [4]. Additionally, Deer et al. [4] found that only 40%-50% of patients receive meaningful pain relief from SCS, while Sdrulla et al. [2] reported that 32% of patients felt > 50% reduction in pain following the procedure. 

DRGS specifically stimulates the dorsal root ganglion (DRG) and its associated dermatome without affecting other tracts in the dorsal columns as in SCS. DRGS was suggested to be a more effective form of treatment for CRPS by Deer et al. [4], producing more meaningful pain relief in patients when compared to SCS alone. Morgalla and colleagues performed a prospective study of 62 patients that was shown to greatly improve pain for CRPS as well as focal neuropathic pain and radiculopathy, suggesting that DRGS is not only more effective for CRPS than SCS but may be more widely applicable than previously thought [5-6]. In contrast, we report a series of DRGS patients with a similar range of focal neuropathic pain who had lower rates of meaningful pain relief than what has been reported in the literature.

## Materials and methods

Between September 2018 and June 2020 DRGS trial was performed on 18 patients for neuropathic pain in multiple areas (neck, back, arms, legs, feet) or CRPS. Charts were reviewed retrospectively and ≥ 50% reduction of pain was used as an outcome measure for the DRGS trial. If trial results were positive, permanent implantation of the DRGS was indicated. Patients with permanent DRGS were separated by positive and negative pain outcomes, defined by whether the patients reported a ≥ 50% reduction of pain, then listed with their co-morbidities to determine potential predictors of negative outcomes.

Inclusion criteria

Patients that had neuropathic pain located in the low back and below, i.e. the low back, pelvis, buttocks, thighs, and calves which were refractory to more conservative treatments and SCS trial as well as a successful DRGS trial were candidates for permanent DRGS.

## Results

Eighteen patients had successful DRGS trials. Success was defined as a ≥ 50% pain reduction, as well as an increased ability to perform daily activities. Of the 18 patients, seven patients elected to not proceed with permanent DRGS. Thus, this study reviews the outcomes of the remaining 11 patients (Tables 1-2). The pain distribution of the 11 remaining patients was as follows: low back pain, upper leg pain, groin pain, lower leg pain, and bilateral lower leg pain. Of the 11 patients that remained for follow-up, four patients reported being completely pain-free with the resumption of normal activities four months post-operation and one reported a significant increase in pain improvement four months post-operation. Of most concern, six patients reported pain ≥ 50% of their pre-surgical pain 4-12 months following the surgery (Table 1). Of the six patients, one patient underwent revision surgery of the malfunctioning lead at five months post-operation with follow up pending, one patient experienced pain following surgery and reprogramming as well as depression secondary to the pain, two patients reported that there was no difference in the pain with the DRGS, and two patients got the DRGS removed. In our data set, smoking had the highest sensitivity and specificity in predicting negative outcomes with a two-proportion z-test score of 5.09 (> 1.96 at 5% alpha level) leading us to reject the null hypothesis which stated that the outcome for the two groups is the same regardless of smoking status. Cancer, diabetes, and age > 70 years, while correlated to worse outcomes, were less sensitive measures for predicting negative outcomes with statistically insignificant z-test scores (< 1.96 at 5% alpha level) of 0.46, 1.86, and 1.86, respectively. 

**Table 1:** Comorbidities and pain distribution of patients with ≤ 50% pain reduction

Patient	1	2	3	4	5	6
Pain distribution	Low back and waist pain	Right lower calf pain	Low back, buttock, and right groin pain	Bilateral upper leg pain	Bilateral low back, pelvic, thigh, and feet pain	Pelvic, left lower back, buttock, and groin pain
Diabetes mellitus	Yes	No	No	Yes	Yes	No
Smoking/tobacco	Yes	Yes	Yes	Yes	No	Yes
Cancer	No	Yes	No	Yes	No	No
Age	59	78	58	70	85	69

**Table 2:** Comorbidities and pain distribution of patients with ≥ 50% pain reduction

Patient	7	8	9	10	11
Pain distribution	Bilateral lower leg neuropathy	Lower left leg pain	Bilateral legs and feet	Low back, bilateral leg to feet, foot numbness	Low mid back pain
Diabetes mellitus	No	No	No	No	No
Smoking/tobacco	Yes	No	Yes	No	No
Cancer	No	No	No	No	No
Age	59	67	67	49	46

## Discussion

In Germany, DRGS was previously reported by Morgalla et al. to be very effective for pain in the leg (76% improvement) and back (44% improvement) at one year. However, we report that DRGS had more modest results with only 45% reporting ≥ 50% reduction in pain and worsening pain in some of those without relief. We suggest that smoking more so than age, diabetes, and cancer may account for the discrepancy in pain relief. Morgalla et al. reported that the average age of their patients was around 56.8 years old. We found our average patient age to be around 64.27 years old. However, Morgalla et al. did not report co-morbidities of their patients in their study, thus we used national rates to compare [5]. The CDC reported diabetes rates of 10% in the US and the WHO reported diabetes rates of 7.4% [7-8]. In our study, we found that 27% of our 11 patients have diabetes and that 50% of patients with ≤ 50% pain reduction have diabetes. Similarly, 18% of our 11 patients have cancer, and 33% of patients with ≤ 50% pain reduction have cancer. In contrast, none of the patients with ≥ 50% reduction in pain have cancer or diabetes. Thus, although not supported by our statistical analyses, there appears to be a modest correlation between our outcomes and these two factors. 

It is important to note that diabetes can also lead to peripheral neuropathy. This might lead to a potential confounding variable as painful diabetic peripheral neuropathy (PDPN) is due to different pathophysiology than the neuropathic back pain evaluated in this study. PDPN is thought to be pain arising as a direct consequence of abnormalities in the peripheral somatosensory system in people with diabetes. These abnormalities are ascribed to symmetrical, length-dependent sensorimotor polyneuropathy attributable to metabolic and microvessel alterations as a result of chronic hyperglycemia exposure [9]. Similarly, lower back pain is also known to have a nociceptive as well as a neuropathic component. The pathophysiology of back pain is complex and nociceptive, and neuropathic pain-generating mechanisms are thought to be involved. Large epidemiological studies show that 20% to 35% of patients with back pain suffer from a neuropathic pain component [10] which is distinct from the pain due to damage from diabetes. However, the neuropathic component of chronic lower back pain is often both under-recognized and undertreated [11]. 

Taking a more whole-person approach, we speculate that psychological factors involved with pain may play a role in false-positive DRGS trials leading to failed DRGS implantation. It has been well documented that psychological factors such as previous anxiety or depression influence the presence and magnitude of chronic pain [12]. Stress-induced analgesia may lead patients to feel that the DRGS trial is working when in reality there is no pain reduction. Stress-induced analgesia has been known for some time to decrease the sensation of pain in people under duress [13], an example being the decreased pain that athletes experience during a competitive contest. The patients undergoing the DRGS trial are usually patients that have pain that has been refractory to several other treatments. In consequence, they may see this treatment as being their last hope for relief from their chronic pain. We believe it to be possible that this stress and anxiety of having refractory chronic pain contributed to some stress-induced analgesia during the seven-day DRGS trial. 

Areas of future study

Areas of further study include larger prospective studies of patients with permanent DRGS implants as well as comparison and analysis of the comorbidities between patients with positive and negative outcomes. Further, multivariate regression with a larger cohort would more substantially determine the independent impact of each comorbidity on negative outcomes of DRGS. Additionally, a study detailing why patients that respond positively to the DRGS trial suffer from negative outcomes following permanent DRGS may also be appropriate. 

Other areas of study include pathophysiologies of how comorbidities such as diabetes mellitus, smoking, cancer, and age interact with the DRGS implant. Literature concerning DRGS and its interactions with diabetic patients is already underway but seems limited to mostly animal models and case reports [14]. Chapman et al. reported complete resolution of symptoms of a patient with painful diabetic neuropathy during a seven-day DRGS trial [15]. However, this was a DRGS trial similar to the one used by the current study and thus shares a similar risk of false positives. Possible reasons for false positives during the DRGS trial have already been previously outlined. Instead, we hypothesize that damaged axons from diabetic endoneurial arteriole hyalinization may cause aberrant firing of the DRG and interfere with the beneficial effects of the DRGS following permanent implantation. Smoking has additionally been noted to have a synergistic effect on stress-induced analgesia which may be further evidence of its potential as a test for negative outcomes of permanent DRGS implantation [16]. Notably, smoking is also a risk factor for developing diabetic neuropathy which may impact permanent DRGS implantation, which is one possible pathophysiology for which has been outlined above [17]. 

Limitations

Limitations of this study are mostly those that are intrinsic to a retrospective case series. These consist of a small sample size which limits the value of any correlation that was drawn. Due to its retrospective nature, the results of this study depend greatly on the accuracy as well as acceptability of the medical records.

## Conclusions

In our case series, we found that there was a discrepancy between DRGS trial outcomes and outcomes following permanent implantation. We found that a correlation may exist between worse outcomes and smoking. Older age, the presence of diabetes, and cancer had more modest associations. These comorbidities may have value as tests for predicting negative outcomes of permanent DRGS implantation. Additionally, we hypothesized that this could also be due to the presence of psychological factors which obscure the true result of the DRGS trial. Thus, we suggest that DRGS be prescribed with caution in these patient populations, and use comorbidities to test for the likelihood of negative outcomes.
